# Ambient ozone and asthma hospital admissions in Texas: a time-series analysis

**DOI:** 10.1186/s40733-017-0034-1

**Published:** 2017-08-01

**Authors:** Julie E. Goodman, Ke Zu, Christine T. Loftus, Ge Tao, Xiaobin Liu, Sabine Lange

**Affiliations:** 1 Gradient, 20 University Road, Cambridge, MA 02138 UK; 2 Gradient, 600 Stewart Street, Suite, Seattle, WA 1900 USA; 3 Texas Commission on Environmental Quality, 12100 Park 35 Circle, Austin, TX USA

**Keywords:** Ozone, Air pollution, Asthma hospital admissions, Epidemiology, Time series

## Abstract

**Background:**

Many studies have evaluated associations between asthma emergency department (ED) visits, hospital admissions (HAs), and ambient ozone (O_3_) across the US, but not in Texas. We investigated the relationship between O_3_ and asthma HAs, and the potential impacts of outdoor pollen, respiratory infection HAs, and the start of the school year in Texas.

**Methods:**

We obtained daily time-series data on asthma HAs and ambient O_3_ concentrations for Dallas, Houston, and Austin, Texas for the years 2003–2011. Relative risks (RRs) and 95% confidence intervals (CIs) of asthma HAs per 10-ppb increase in 8-h maximum O_3_ concentrations were estimated from Poisson generalized additive models and adjusted for temporal trends, meteorological factors, pollen, respiratory infection HAs, day of the week, and public holidays. We conducted a number of sensitivity analyses to assess model specification.

**Results:**

We observed weak associations between total asthma HAs and O_3_ at lags of 1 day (RR_10 ppb_ = 1.012, 95% CI: 1.004–1.021), 2 days (RR_10 ppb_ = 1.011, 95% CI: 1.002–1.019), and 0–3 days (RR_10 ppb_ = 1.017, 95% CI: 1.005–1.030). The associations were primarily observed in children aged 5–14 years (e.g., for O_3_ at lag 0–3 days, RR_10 ppb_ = 1.037, 95% CI: 1.011–1.064), and null in individuals 15 years or older. The effect estimates did not change significantly with adjustment for pollen and respiratory infections, but they attenuated considerably and lost statistical significance when August and September data were excluded. A significant interaction between time around the start of the school year and O_3_ at lag 2 day was observed, with the associations with pediatric asthma HAs stronger in August and September (RR_10 ppb_ = 1.040, 95% CI: 1.012–1.069) than in the rest of the year (October–July) (RR_10 ppb_ = 1.006, 95% CI: 0.986–1.026).

**Conclusions:**

We observed small but statistically significant positive associations between total and pediatric asthma HAs and short-term O_3_ exposure in Texas, especially in August and September. Further research is needed to determine how the start of school could modify the observed association between O_3_ and pediatric asthma HAs.

**Electronic supplementary material:**

The online version of this article (doi:10.1186/s40733-017-0034-1) contains supplementary material, which is available to authorized users.

## Background

Asthma is a chronic lung disease characterized by inflammation and narrowing of the conducting airways. Acute exacerbations can be triggered by a variety of factors, including stress, physical exertion, airborne allergens, respiratory infections, and both indoor and outdoor air pollutants [1, 2]. Asthma exacerbations can be life-threatening if not treated and are one of the most common causes of emergency department (ED) visits and hospital admissions (HAs) in the US.

Numerous time-series and case-crossover epidemiology studies have been conducted to evaluate whether incidence rates of asthma ED visits and HAs increase following short-term increases in regional ozone (O_3_) concentrations. These studies have been conducted in a variety of locations across North America, and include analyses of ED visits for asthma in New York City [3], Atlanta [4], Seattle [5], and Canada [6, 7], and analyses of asthma HAs in New York City [8] and Seattle [9]. Most of these studies reported small, statistically significant elevations of population-average ED visits and HAs for asthma in the days following elevated O_3_ levels, and some evidence indicates that certain groups are more susceptible to O_3_ exposure, including children [8] and the elderly [10].

In Texas, where O_3_ is monitored on a year-round basis, O_3_ levels have sometimes exceeded the National Ambient Air Quality Standard (NAAQS) in Dallas-Fort Worth, Houston-Galveston-Brazoria, and Austin areas from 2003 to 2011 [11]. For example, in 2009–2011, the annual fourth highest daily maximum 8-h average O_3_ concentrations at the majority of monitoring sites in the Dallas-Fort Worth and Houston-Galveston areas exceeded the then-current O_3_ NAAQS of 75 parts per billion (ppb). Between 2007 and 2010, Dallas County and Harris County also reported the highest number of HAs for asthma in Texas [12].

Despite elevated O_3_ concentrations and the relatively high burden of asthma in Dallas and Houston, to our knowledge, no studies have measured associations between *daily* O_3_ exposure and either HAs or ED visits for asthma in any regions of Texas. Thus, we conducted a time-series study of ambient O_3_ and HAs for asthma in Dallas, Houston, and Austin, the three metropolitan areas for which pollen data are available, with stratification by patient age, to determine whether associations vary with age. Because time-series analyses of short-term effects of air pollution can be confounded by time-varying factors associated with both exposure and outcome, we aimed to thoroughly investigate several potential factors known to affect the risk of asthma exacerbations, including outdoor pollen and other aeroallergens, respiratory infection HAs, and – for children – the return to school in August and September. Finally, our analytic approach included a rigorous assessment of model specification, as previous research has demonstrated that results of time-series studies are sensitive to statistical modeling approaches [13, 14].

## Methods

### Hospital admission data

We obtained daily hospital discharge data from 2003 to 2011 from the Texas Department of State Health Services (TDSHS), for patients who were hospitalized in emergency or urgent care departments in Houston, Dallas, or Austin with a primary diagnosis of asthma (International Classification of Disease, 9th Revision [ICD-9] code 493). Because asthma diagnoses in younger children are not reliable [15], we excluded patients who were younger than 5 years old.

Our study was reviewed and approved by the TDSHS Institutional Review Board (IRB).

### Ambient ozone data

The Texas Commission on Environmental Quality (TCEQ) maintains ambient air monitoring stations, where ambient O_3_ concentrations are monitored year round. We obtained ambient O_3_ monitoring data from 2003 to 2011 for three metropolitan areas – Dallas (8 monitoring sites), Houston (44 monitoring sites), and Austin (6 monitoring sites) – from TCEQ. We calculated site-specific daily maximum 8-h moving average O_3_ concentrations and averaged over all sites within each area to obtain area-specific daily average 8-h maximum O_3_ concentrations over the entire time period.

### Meteorological data

We obtained daily mean temperatures and daily dew point temperatures, which were also measured at air monitoring stations, for the same time period and areas from TCEQ. We calculated area-level daily averages of these meteorological variables.

### Pollen data

The National Allergen Bureau (NAB), a section of the American Academy of Allergy, Asthma and Immunology (AAAAI), provides aeroallergen levels from a network of counting stations throughout the US. We obtained the data on daily total pollen and aeroallergen counts from the Allergy and Asthma Center in Georgetown (Austin) from NAB, and we obtained the data for Dallas (ENTDocs Ear, Nose, Throat & Allergy Testing & Treatment in Plano; Family Allergy and Asthma Care in Flower Mound) and Houston (Station 2) directly from the individual stations. Because there are no data available for weekends and holidays, we imputed the missing values as described by Gleason et al. [16]. Briefly, a day with missing pollen counts was assigned the pollen counts from the previous day for up to three consecutive days with missing values. If there were more than three consecutive days with missing pollen values, the remainder of the days were given the pollen value from the succeeding day.

### Statistical analysis

Based on patient residential zip code, we aggregated individual-level data to metropolitan area-level daily counts of total asthma HAs, as well as asthma HAs for three specific age groups (5–14 years, 15–64 years, and 65 years and older) in Houston, Dallas, and Austin.

We used a Poisson Generalized Additive model to estimate the relative risks (RRs) and 95% confidence intervals (CIs) for asthma HAs associated with a 10-ppb increase in ambient O_3_ concentrations at various lags, including single-day lags from current day to six days prior (lag 0, 1, 2, 3, 4, 5, and 6), and multiple-day lags of day 0 to 3 days prior (lag 0–3) and day 0 through six days prior (lag 0–6). For each area, we included the natural logarithm of population size as an offset term and the counts of asthma HAs in the previous four days as covariates to account for autocorrelations in the dependent variable. We adjusted for temporal trend, immediate and delayed effects of meteorological factors, day of the week, and public holidays. We combined the area-specific effect estimates using a random effects model. No significant overdispersion was observed in the time-series data.

The main model is:

$$ \mathit{\log}\left[E\left({Y}_t\right)\right]=\mathit{\log}\left({Pop}_j\right)+\alpha +\beta \times {Ozone}_t+\gamma \times {DOW}_t+\xi \times {Holiday}_t+{Area}_j+ ns\left({time}_t,\frac{8}{year}\right)+ ns\left(Tem{p_{lag0}}_t,3\right)+ ns\left(Tem{p_{lag13}}_t,3\right)+ ns\left(De{w_{lag0}}_t,3\right)+ ns\left(De{w_{lag13}}_t,3\right)+{\upsilon}_1\times {Y}_{t-1}+{\upsilon}_2\times {Y}_{t-2}+{\upsilon}_3\times {Y}_{t-3}+{\upsilon}_4\times {Y}_{t-4}, $$

where.

*E*(*Y*_*t*_) = the expected count of asthma HAs on day *t*.

*Pop*_*j*_ = the total population of the *j*th area

*Ozone*_*t*_ = the O_3_ concentrations on day *t*

*DOW*_*t*_ = the categorical variable with seven levels specifying the day of the week on day *t*

*Holiday*_*t*_ = the indicator variable specifying whether day *t* is a federal holiday

*Area*_*j*_ = a random intercept for area j

*ns*(*time*_*t*_, 8 *df*/*year*) = a cubic spline function of time with eight knots per year to control for temporal trends

*ns*(*Temp*_*lag*0*t*_, 3) = a cubic spline function of the current-day average temperature with 3 degrees of freedom

*ns*(*Temp*_*lag*13*t*_, 3) = a cubic spline function of the average of lag 1 through 3 day temperature with 3 degrees of freedom

*ns*(*Dew*_*lag*0*t*_, 3) = a cubic spline function of the current-day dew point with 3 degrees of freedom

*ns*(*Dew*_*lag*13*t*_, 3) = a cubic spline function of the average of lag 1 through 3 day dew point with 3 degrees of freedom

*Y*_*t* − 1_, *Y*_*t* − 2_, *Y*_*t* − 3_, and *Y*_*t* − 4_ = lag 1, lag 2, lag 3, and lag 4 day of asthma HA counts on day *t*.

We evaluated pollen levels as a potential confounder for the O_3_-asthma association. Daily pollen counts were added into the main model in different forms. First, we created a categorical variable (0, <25th percentile, ≥25th percentile and <50th percentile, ≥50th percentile and <75th percentile, ≥75th percentile). Second, we log-transformed daily pollen counts to improve normality (Additional file 1 Fig. S1) and included log(pollen counts) in the model as a linear term. Third, we used the cubic spline of daily pollen counts with 3 degrees of freedom. Similar approaches were used for aeroallergen counts (Additional file 1 Fig. S2). We evaluated various lags of pollen and aeroallergen counts, including same day (lag 0), previous day (lag 1), two days prior (lag 2), and the average of 0 to 2 days prior (lag 0–2). Because there are two counting stations for Dallas, we used the data from the Plano station in the main analysis and the data from the Flower Mound station for sensitivity analyses.

In addition to pollen, we also evaluated whether respiratory infection HAs would confound the association between ambient O_3_ and asthma HAs. We identified patients with primary diagnoses of respiratory infections (pneumonia, ICD-9 codes 480–486; upper respiratory infection, ICD-9 codes 460–465; influenza, ICD-9 code 487) and generated area-level daily counts of total respiratory infections. We evaluated the short-term effect of respiratory infection HAs by adding the same-day total respiratory infection HAs (lag 0) and the average of lag 0 through 2 day total respiratory infection HAs (lag 0–2) into the model as either a linear covariate or cubic splines with 3 degrees of freedom.

We also examined whether the start of the school year would impact the association between ambient O_3_ and asthma HAs. We first excluded days from August and September and estimated risks of total asthma HAs and pediatric asthma HAs for children aged 5 to 14 years. We also created an indicator variable for the two months of August and September and included the indicator variable and/or the product terms of this indicator variable and the O_3_ variables in regression models to evaluate confounding and effect modification.

We conducted a series of sensitivity analyses to examine whether specifications of models and covariates impact the results. First, pollen data from the Flower Mound station, instead of Plano, were used for Dallas. Second, we used different degrees of freedom per year for the temporal splines. Third, we used different degrees of freedom splines of temperature and dew point. To evaluate whether the model would give rise to spurious associations, we conducted two sensitivity analyses: 1) including O_3_ concentrations at lag −1 day in the model, and 2) using a health endpoint that is unrelated to ambient air pollution (acute appendicitis, ICD-9 code 540) as the dependent variable. Last, we repeated all analyses with total aeroallergen (pollen and mold) counts instead of pollen counts. Only two metropolitan areas, Dallas and Houston, were included in this sensitivity analysis because data on mold were not available for Austin.

All statistical analyses were conducted using SAS 9.4 (SAS Institute Inc., Cary, NC).

## Results

There were a total of 74,824 asthma HAs in Dallas, Houston, and Austin, Texas from 2003 to 2011, which included 16,879 patients 5 to 14 years of age (mean ± standard deviation [SD]: 1.7 ± 2.0 daily visits), 40,991 who were 15 to 64 years of age (4.2 ± 3.0 daily visits), and 16,954 who were 65 years of age or older (1.7 ± 1.6 daily visits). The mean area-level 8-h maximum ambient O_3_ concentration for all three metropolitan areas was 41.8 ppb. Daily pollen counts were on average about 300, with a wide range from 0 to over 30,000. The average temperature was 68.1 °F (range: 16.6–97.7), and the average dew point temperature was 55.1 °F (range: 6–76.8). Correlations between these factors were generally weak but highly statistically significant. O_3_ appeared to be positively correlated with temperature (*r* = 0.3) and pollen counts (*r* = 0.2), and negatively correlated with asthma HAs (*r* = −0.1). Descriptive statistics and pairwise correlations for asthma HAs, ambient O_3_, pollen, and meteorological factors are presented in Tables 1 and 2. Among the three metropolitan areas, ambient O_3_ concentrations and meteorological factors were comparable, but Dallas had lower average daily pollen counts than Austin and Houston (Additional file 1 Table S1).

**Table 1:** Daily asthma HAs, ambient O_3_ concentrations, pollen counts, and meteorological variables in Dallas, Houston, and Austin, Texas, from 2003 to 2011

Variable name	N of days with data	Mean	SD	Minimum	10^th^ Percentile	25^th^ Percentile	Median	75^th^ Percentile	90^th^ Percentile	Maximum
Daily asthma HAs, all ages (N)	9861	7.6	5.1	0	2	3	7	11	15	32
Daily asthma HAs, 5–14 years (N)	9861	1.7	2.0	0	0	0	1	3	4	17
Daily asthma HAs, 15–64 years (N)	9861	4.2	3.0	0	1	2	4	6	8	19
Daily asthma HAs, 65+ years (N)	9861	1.7	1.6	0	0	0	1	3	4	12
Daily 8-h maximum O_3_ (ppb)	9839	41.8	14.5	2.0	24.9	31.3	39.7	51.3	62.3	107.0
Daily total pollen counts (N)	9861	283.5	1007.6	0	0	3	26	163	664	30,961
Temperature (^°^ *F*)	9840	68.1	14.3	16.6	47.2	57.9	70.3	80.1	84.6	97.7
Dew point temperature (^°^ *F*)	9845	55.1	15.4	6	31.6	43.5	59.5	68.2	72	76.8

*HA* hospital admission, *ppb* parts per billion, *SD* standard deviation, *O*_*3*_ ozone

**Table 2:** Spearman correlation coefficients for total daily asthma HAs, ambient O_3_ concentrations, pollen counts, and meteorological variables in Dallas, Houston, and Austin, Texas, from 2003 to 2011

Correlation coefficients (*p*-value)	Asthma HAs	Average temperature	Dew point	Daily 8-h maximum O_3_	Daily total pollen counts
Asthma HAs	1	−0.3	−0.2	−0.1	0.1
		(<0.0001)	(<0.0001)	(<0.0001)	(<0.0001)
Average temperature	−0.3	1	0.9	0.3	−0.2
	(<0.0001)		(<0.0001)	(<0.0001)	(<0.0001)
Dew point	−0.2	0.9	1	0.1	−0.2
	(<0.0001)	(<0.0001)		(<0.0001)	(<0.0001)
Daily 8-h maximum O_3_	−0.1	0.3	0.1	1	0.2
	(<0.0001)	(<0.0001)	(<0.0001)		(<0.0001)
Daily total pollen counts	0.1	−0.2	−0.2	0.2	1
	(<0.0001)	(<0.0001)	(<0.0001)	(<0.0001)	

*HA* hospital admission, *O*_*3*_ ozone

Adjusted RRs for total and age-specific asthma HAs per 10-ppb increase in ambient O_3_ concentrations at various lags are presented in Table 3. For total asthma HAs, the association with same-day 8-h maximum O3 concentrations was null (RR = 1.007; 95% CI: 0.998–1.015). Total asthma HAs were positively associated with O_3_ concentrations the previous day (lag 1) (RR = 1.012; 95% CI: 1.004–1.021) and two days prior (lag 2) (RR = 1.011; 95% CI: 1.002–1.019), and with the average of O_3_ concentrations 0 to 3 days prior (lag 0–3) (RR = 1.017; 95% CI: 1.005–1.030). The effect of O_3_ on pediatric asthma HAs was stronger, with statistically significant associations observed for all three single-day lags (O_3_ at lag 0 day: RR = 1.027, 95% CI: 1.008–1.047; lag 1 days: RR = 1.025, 95% CI: 1.007–1.043; lag 2 day: RR = 1.019, 95% CI: 1.001–1.038) and the multiple-day lag (O_3_ at lag 0–3 days: RR = 1.037, 95% CI: 1.011–1.064). In contrast, O_3_ was not statistically significantly associated with asthma HAs in patients aged 15 years or older for most lag periods. Total and age-specific asthma HAs were generally not statistically significantly associated with O_3_ concentrations at more than two days prior to the outcome, i.e., lag 3, 4, 5, 6 day and lag 0–6 days(Additional file 1 Table S2).

**Table 3:** Relative risks^a^ of asthma HAs per 10 ppb increase in daily 8-h maximum O_3_ concentrations in Dallas, Houston, and Austin, Texas, from 2003 to 2011

Asthma HAs	Same day (lag 0)		1-day lag (lag 1)		2-day lag (lag 2)		4-day average (lag 0–3)	
	RR^a^ (95% CI)	*p*-value	RR^a^ (95% CI)	*p*-value	RR^a^ (95% CI)	*p*-value	RR^a^ (95% CI)	*p*-value
All ages	1.007 (0.998–1.015)	0.15	1.012 (1.004–1.021)	0.004	1.011 (1.002–1.019)	0.01	1.017 (1.005–1.030)	0.005
5–14 years	1.027 (1.008–1.047)	0.01	1.025 (1.007–1.043)	0.01	1.019 (1.001–1.038)	0.03	1.037 (1.011–1.064)	0.01
15–64 years	1.005 (0.993–1.017)	0.41	1.011 (1.000–1.023)	0.05	1.005 (0.994–1.016)	0.39	1.015 (0.999–1.032)	0.07
65+ years	0.996 (0.977–1.014)	0.64	1.004 (0.986–1.022)	0.67	1.016 (0.998–1.034)	0.07	1.005 (0.980–1.031)	0.69

*HA* hospital admission, *ppb* parts per billion, *RR* relative risk, *O*_*3*_ ozone, *CI* confidence interval

^a^RRs were estimated from Poisson generalized additive models and adjusted for cubic splines of calendar time (8 d.f. per year), cubic splines of same-day temperature (3 d.f.), cubic splines of the average of lag 1 through 3 day temperature (3 d.f.), cubic splines of same-day dew point (3 d.f.), cubic splines of the average of lag 1 through 3 day dew point (3 d.f.), day of the week, and public holidays

We evaluated daily pollen levels and total respiratory infection HAs as potential confounders for the associations between ambient O_3_ concentrations and total and pediatric asthma HAs (Table 4). Adjustment for daily pollen counts alone (Model 2), or in combination with total respiratory infection HAs (Model 3), did not impact the effect estimates for O_3_ in any meaningful way. The generally null associations between ambient O_3_ concentrations and asthma HAs for patients aged 15 years or older were not changed with the adjustment for pollen counts and total respiratory infection HAs (data not shown). Using a linear term, categorical variables, or cubic splines to specify pollen counts had little impact on the risk estimates of O_3_ (Additional file 1 Table S3). Using pollen data from a different counting station for Dallas also did not change the results significantly (Additional file 1 Table S4). Pollen counts and total respiratory infection HAs expressed with a linear term, as a categorical variable, or cubic splines at various lags were evaluated, but the observed confounding effects by these two factors were minimal at all lags (Additional file 1 Table S5). Using total aeroallergen counts instead of pollen counts did not result in any greater changes in the risk estimates for O_3_ (Additional file 1 Table S6).

**Table 4:** Relative risks of asthma HAs per 10-ppb increase in daily 8-h maximum O_3_ concentrations, with adjustments for pollen and respiratory infections, in Dallas, Houston, and Austin, Texas from 2003 to 2011

O_3_ concentrations	Model 1^a^		Model 2^b^		Model 3^c^	
	Full year	October–July	Full year	October–July	Full year	October–July
*Asthma HAs, all ages*						
Same day	1.007 (0.998–1.015)	0.999 (0.988–1.009)	1.005 (0.997–1.014)	0.998 (0.987–1.008)	1.005 (0.996–1.014)	0.997 (0.986–1.008)
Lag 1 day	1.012 (1.004–1.021)	1.011 (1.001–1.021)	1.011 (1.003–1.020)	1.010 (1.000–1.021)	1.010 (1.001–1.018)	1.008 (0.998–1.018)
Lag 2 day	1.011 (1.002–1.019)	1.008 (0.998–1.018)	1.010 (1.002–1.019)	1.008 (0.998–1.018)	1.008 (1.000–1.016)	1.005 (0.995–1.015)
Lag 0–3 days	1.017 (1.005–1.030)	1.011 (0.996–1.026)	1.016 (1.004–1.028)	1.010 (0.995–1.025)	1.013 (1.001–1.025)	1.006 (0.991–1.021)
*Asthma HAs, 5–14 years*						
Same day	1.027 (1.008–1.047)	1.014 (0.991–1.039)	1.024 (1.005–1.044)	1.013 (0.989–1.037)	1.023 (1.004–1.043)	1.012 (0.988–1.037)
Lag 1 day	1.025 (1.007–1.043)	1.018 (0.996–1.041)	1.023 (1.005–1.041)	1.018 (0.996–1.041)	1.022 (1.004–1.040)	1.016 (0.994–1.039)
Lag 2 day	1.019 (1.001–1.038)	0.999 (0.977–1.021)	1.018 (1.0002–1.036)	0.999 (0.977–1.021)	1.016 (0.998–1.034)	0.997 (0.975–1.019)
Lag 0–3 days	1.037 (1.011–1.064)	1.011 (0.978–1.045)	1.034 (1.008–1.060)	1.010 (0.977–1.044)	1.031 (1.005–1.058)	1.007 (0.974–1.041)

*HA* hospital admission, *O*_*3*_ ozone

^a^Model 1 adjusted for cubic splines of calendar time (8 d.f. per year), cubic splines of same-day temperature (3 d.f.), cubic splines of the average of lag 1 through 3 day temperature (3 d.f.), cubic splines of same-day dew point (3 d.f.), cubic splines of the average of lag 1 through 3 day dew point (3 d.f.), day of the week, and public holidays

^b^Model 2 was Model 1 with additional adjustment for total pollen counts (lag 0–2 days, categorical)

^c^Model 3 was Model 1 with additional adjustment for total pollen counts (lag 0–2 days, categorical) and total respiratory infection HAs (lag 0–2 days, cubic splines with 3 d.f.)

We also considered the impact of the start of the school year on the observed associations between ambient O_3_ and total and pediatric asthma HAs (Table 4). Excluding data from August and September, the RRs for O_3_ and total asthma HAs were attenuated and generally did not remain statistically significant. Similarly, associations between O_3_ and pediatric asthma HAs became null when data around the start of school year were excluded from the analyses.

We further evaluated whether the time period around the start of school year confounded and/or modified the associations between pediatric asthma HAs and ambient O_3_ concentrations (Table 5). Inclusion of an indicator variable for the two months of August and September resulted in only minimal changes in the risk estimates for ambient O_3_ concentrations, suggesting that this time period did not confound the observed association between pediatric asthma HAs and ambient O_3_. Results from Model 3 show that the effect estimates of ambient O_3_ concentrations at lag 2 day were considerably stronger in August and September (RR = 1.040, 95% CI: 1.012–1.069) than those in the rest of the year (October–July) (RR = 1.006, 95% CI: 0.986–1.026). The interactions, however, were not significant for same-day concentrations (p_interaction_ = 0.24), previous-day concentrations (p_interaction_ = 0.75), and concentrations at lag 0–3 days (p_interaction_ = 0.12). This finding suggests that the association between pediatric asthma HAs and ambient O_3_ at lag 2 day was modified by the time period around the start of the school year.

**Table 5:** Relative risks of pediatric asthma HAs (5–14 years) per 10-ppb increase in daily 8-h maximum O_3_ concentrations, stratified by time period, with adjustments for pollen and respiratory infections, in Dallas, Houston, and Austin, Texas from 2003 to 2011

O_3_ concentrations	Model 1^a^	Model 2^b^	Model 3^c^	
	Full year	Full year	August–September	October–July
Same day	1.023 (1.004–1.043)	1.023 (1.004–1.043)	1.036 (1.007–1.066)	1.017 (0.995–1.039)
			P_interaction_ = 0.24	
Lag 1 day	1.022 (1.004–1.040)	1.021 (1.003–1.040)	1.025 (0.997–1.054)	1.020 (0.999–1.041)
			P_interaction_ = 0.75	
Lag 2 day	1.016 (0.998–1.034)	1.016 (0.998–1.034)	1.040 (1.012–1.069)	1.006 (0.986–1.026)
			P_interaction_ = 0.03	
Lag 0–3 days	1.031 (1.005–1.058)	1.031 (1.005–1.057)	1.053 (1.015–1.093)	1.020 (0.991–1.050)
			P_interaction_ = 0.12	

*HA* hospital admission, *ppb* parts per billion, *SD* standard deviation, *O*_*3*_ ozone

^a^Model 1 adjusted for cubic splines of calendar time (8 d.f. per year), cubic splines of same-day temperature (3 d.f.), cubic splines of the average of lag 1 through 3 day temperature (3 d.f.), cubic splines of same-day dew point (3 d.f.), cubic splines of the average of lag 1 through 3 day dew point (3 d.f.), day of the week, public holidays, total pollen counts (lag 0–2 days, categorical), and total respiratory infection HAs (lag 0–2 days, cubic splines with 3 d.f.)

^b^Model 2 was Model 1 with additional adjustment for the start of school year (indicator variable for August/September)

^c^Model 3 was Model 1 with additional adjustment for the start of school year (indicator variable for August/September) and the interaction between the start of school year and ambient O_3_ concentrations

Finally, we conducted a series of sensitivity analyses regarding model and covariate specifications. We found that the results were not sensitive to increased degrees of freedom in cubic splines of temperature and dew point (Additional file 1 Table S7). We also evaluated whether assuming 4, 8, or 12 degrees of freedom in the temporal splines impacted results for all ages combined or specific age categories, and for lags of 0, 1, 2, and 0–3 days. We found that effect estimates for O_3_ were higher when assuming 4 degrees of freedom vs. 8, but the results were similar for 8 and 12 degrees of freedom (Additional file 1 Table S8). O_3_ concentrations at lag −1 day were not associated with asthma HAs (Additional file 1 Table S9), nor were acute appendicitis HAs associated with ambient O_3_ levels (Additional file 1 Table S10), indicating that the main model did not give rise to spurious associations.

## Discussion

In an all-year time-series analysis of ambient O_3_ and asthma HAs in three major metropolitan regions of Texas, we found small, statistically significant increases in daily rates of total asthma HAs one to 3 days following elevated O_3_ levels. Associations were strongest for the 5- to 14-year-old age group, but null for people over the age of 14. The observed effects of ambient O_3_ on asthma HAs in school-age children appeared to be stronger in the time period around the start of school year (August and September) but were null in the rest of the year (October–July). In sensitivity analyses, we evaluated whether the strength and precision of estimates varied with differences in model specification, and we found that our results were robust to most variations. It is unknown whether any such model misspecification would affect the validity of associations at shorter lags.

Only a few studies have evaluated O_3_ and asthma exacerbations in Texas. In a study of lifeguards in the Galveston area, Thaller et al. [17] reported that short term O_3_ exposure was associated with a reduced ratio of forced expiratory volume in one second to forced vital capacity (FEV_1_/FVC), with a greater decrease observed for individuals with asthma (change = −0.6, *p* < 0.05) than nonasthmatics (change = −0.4, *p* < 0.01). Zora et al. [18], however, reported that O_3_ was not associated with worsened control of asthma in El Paso, Texas (change in asthma control questionnaire score = 0.006, *p* = 0.9). Raun et al. [19] conducted a study of ambient O_3_ and ambulance-treated asthma attacks in Houston and reported a small elevation in asthma events associated with a 20-ppb increase in O_3_ concentrations at lag 0–2 days (RR = 1.07, 95% CI: 1.03–1.11). These studies varied considerably with regard to methodology and asthma exacerbation endpoints, and reported inconsistent results. The association between ambient O_3_ concentrations and emergency/urgent care asthma HAs observed in our study was similar to the findings of Raun et al. [19], likely due to the similarity between the asthma exacerbation endpoints evaluated.

It is notable that our results are quantitatively similar to those of other time-series and case-crossover investigations of ambient O_3_ and asthma ED visits and HAs conducted in locations across the world. Recently, Zheng et al. [20] conducted a systematic review of all published epidemiology analyses of this topic; pooling the results of 71 studies in a meta-analysis, Zheng et al. [20] estimated a meta-RR of 1.018 (95% CI: 1.013, 1.023) per 10-ppb increase in O_3_ for individuals of all ages. This is comparable to the magnitude of associations with total asthma HAs we observed in our main analyses.

In addition to our core analysis, a primary objective of our investigation was to determine whether observed associations between O_3_ and rates of asthma HAs were affected by various time-varying factors that could confound ecological relationships between air pollution and respiratory health. Because any true associations between air pollution and health are small in absolute magnitude, residual or unmeasured confounding can strongly impact the accuracy of study results [14]. In contrast to observational study designs in which individual-level health, exposure, and covariate data are collected (i.e., panel studies or prospective cohort studies), time-series studies are ecological in design and information on potential confounders is not available for specific individuals. Typically, confounding by time-varying factors is addressed in time-series analyses using flexible splines of calendar time, presumed to act as surrogates for potential confounders that vary on long to moderate time frames, on the order of months, seasons, or years [21].

We assessed whether the association between O_3_ and asthma was affected by accounting for the “September effect,” a spike in rates of asthma exacerbations for school-age children that occurs following the beginning of each new school year [22]. Researchers have ascribed this increase in asthma exacerbations to a variety of factors, including stress [22] or exposure to respiratory viruses [23], and some time-series analyses of pediatric asthma exclude the period at the beginning of the school year to avoid confounding by this factor [3]. Excluding August and September from our analysis substantially affected our results (effect estimates were strongly attenuated and less precise). Further evaluation revealed that the time period around the start of school year was not a confounder, but an effect modifier for the observed associations between ambient O_3_ and asthma HAs among school-age children. Effects of O_3_ appeared to be stronger in August and September compared to the rest of the year. This interaction observed in our study suggests that children are more susceptible to O_3_ during this period (perhaps because of stress or increased viral infections).

We also explored the possibility that observed associations between O_3_ and asthma hospitalizations were confounded by exposure to airborne pollen. Previous research indicates that increased regional concentrations of outdoor aeroallergens are associated with increases in numerous outcomes related to respiratory morbidity, including asthma ED visits [16, 24–26] and asthma hospitalizations [27]. If daily O_3_ concentrations are even slightly correlated with ambient pollen concentrations, measured associations between O_3_ and asthma exacerbations could be confounded. Correlation coefficients between measured O_3_ and pollen for a particular study region may not reflect the direction or magnitude of true correlation in the case that either measurement is affected by random measurement error.

To address the hypothesis that asthma-O_3_ associations were confounded by ambient pollen, we adjusted for concentrations of outdoor pollen measured at regional stations up to two days prior to the date of outcome. We observed minimal impact on the associations between O_3_ and daily asthma HAs for any age group. Based on this finding alone, however, we cannot rule out the possibility that outdoor pollen and other aeroallergens are confounders for the O_3_-asthma association. We used pollen counts measured at stations in Houston, Dallas, and Austin to estimate exposures in the study population, but this approximation may poorly reflect actual pollen exposures. Pollen exposures generally exhibit a high degree of spatial variability across a region and, as a result, the concentrations from local stations may be poor surrogates for true exposure. In addition, airborne pollen is a highly heterogeneous mixture. Only sensitized individuals are likely to experience respiratory effects on days of elevated pollen levels, and, furthermore, the risk of respiratory effects for each sensitized individual varies strongly by type of pollen, because different individuals are sensitized to one or more types of pollen. Therefore, the ecological design of our study, as well as the measurement error associated with estimates of pollen exposure, may limit our model’s ability to accurately account for any true confounding by pollen that may exist. In addition, the shape of the dose-response relationship between pollen exposure and asthma exacerbations, on either the population or individual level, is unknown; one recent analysis indicated that the associations between ambient pollen and respiratory health varies strongly by the form of the pollen covariate included in statistical models [24]. Thus, our finding of limited confounding by pollen in our study region may have occurred because pollen exposure is incorrectly specified in our models.

Similarly, we pursued the hypothesis that confounding could occur in time-series studies of O_3_ and asthma due to the very strong association between respiratory infections and asthma exacerbations. For example, viral infections are the single most important trigger of pediatric asthma exacerbations, estimated to cause over 80% of acute exacerbations [28]. Panel studies and time-series studies of air pollution and asthma have presented some evidence that respiratory infections may confound observed associations [4, 29]. We assessed evidence for any such confounding in our study population by adding adjustment variables derived from daily counts of HAs for respiratory infections. Specifically, we varied lag relationships and functional forms for total respiratory infection HAs for inclusion in statistical models. We observed little evidence that respiratory HAs confounded relationships between O_3_ and total and pediatric asthma HAs in Texas. Previous studies have shown that specific viruses increase risk of asthma exacerbations while others viruses do not [30]. Because respiratory infection HAs were only a crude proxy measure of individual exposure to respiratory infections, our analyses cannot rule out confounding by respiratory infections completely.

There are several strengths of our investigation that should be considered when interpreting our results, including the large sample size in our analysis and the use of area-specific monitoring data to estimate daily O_3_ exposures to populations living in different areas of the study region. We explored confounding by pollen, respiratory infection HAs, and the start of the school year, using an alternative approach to adjusting for flexible functions of calendar time, which is the approach typically used to approximate seasonal changes in time-varying confounders. We also rigorously assessed the robustness of our model specification, including an analysis evaluating the associations between asthma HAs occurring the day before O_3_ exposure [31] and a negative control analysis, in which we evaluated whether acute appendicitis was associated with O_3_ [32]. Results of these sensitivity analyses did not provide evidence for model misspecification; however, these sensitivity analyses cannot completely rule out all forms of model misspecification. The fact that our analyses for extended lag periods consistently indicated a statistically significant decrease in asthma HAs six days following an increase in ambient O_3_ supports the potential for model misspecification, because it is unlikely that O_3_ truly protects against asthma exacerbations six days later.

In addition to the uncertainties in model specification and in estimating true exposure to airborne pollen and respiratory infections, additional limitations in our time-series approach should be acknowledged. Like many other time-series studies, we estimated daily O_3_ exposure using measurements from central site monitors in each metropolitan area, and these estimates may vary substantially from true O_3_ exposures in the study population. The resultant exposure measurement error could be classical and/or Berkson in nature, biasing effect estimates and standard errors in directions that are difficult to predict [33]. Additional data collection would be needed to characterize the precise effect of this error on epidemiology associations; for example, a personal exposure study could be conducted to quantify how well central site monitor measurements predict personal exposure. Similarly, measurement errors are also likely present in the meteorological data. In addition, our analysis did not account for any potential confounding by co-pollutants, such as particulate matter less than 2.5 μm in diameter, that may be correlated with O_3_ in this study area.

## Conclusions

We observed weak, positive associations between ambient O_3_ and asthma HAs in Texas, with the strongest effects among school-age children. However, these associations varied significantly during the year, with the strongest associations observed at the beginning of the school year, a period of large increases in pediatric asthma exacerbations. Further research is needed to determine whether this effect modification occurs in other time-series analyses of pediatric asthma and air pollution.

## Additional file

Additional file 1: Fig. S1. Histograms of pollen counts. A. Original data. B. Log-transformed data. Fig. S2 Histograms of aeroallergen counts. A. Original data. B. Log-transformed data. **Table S1** Descriptive statistics of asthma HAs, ambient O_3_, pollen, and meteorological factors in Dallas, Houston, and Austin, Texas, from 2003 to 2011. **Table S2** Associationsa between asthma HAs and ambient O3 concentrations in Dallas, Houston, and Austin, Texas, from 2003 to 2011. **Table S3** Associationsa between asthma HAs and ambient O3 concentrations, with adjustment for pollen, in Dallas, Houston, and Austin, Texas, from 2003 to 2011. **Table S4** Associations between asthma HAs and ambient O3 concentrations, with adjustment for pollen using alternative dataa for Dallas. **Table S5** Associations between asthma HAs and ambient O3 concentrations, with adjustments for pollen and respiratory infection HAs, in Dallas, Houston, and Austin, Texas, from 2003 to 2011. **Table S6** Results of asthma HAs per 10 ppb increase in O3 concentrations, with adjustment for aeroallergen, in Dallas and Houston, Texas, from 2003 to 2011. **Table S7** Sensitivity analyses with different specifications of temperature and dew point . **Table S8** Sensitivity analysesa with different specifications of temporal trend. **Table S9** Sensitivity analyses^a^ with O_3_ concentrations at lag −1 day. **Table S10** Associationsa between acute appendicitis HAs and ambient O3 in Dallas, Houston, and Austin, Texas, from 2003 to 2011. (XLSX 95 kb)

## Abbreviations

AAAAI: American Academy of Allergy, Asthma and Immunology
CI: Confidence Interval
ED: Emergency Department
FEV_1_: Forced Expiratory Volume in One Second
FVC: Forced Vital Capacity
HA: Hospital Admission
ICD-9: International Classification of Disease, 9th Revision
IRB: Institutional Review Board
NAAQS: National Ambient Air Quality Standard
NAB: National Allergen Bureau
O_3_: Ozone
Ppb: Parts Per Billion
RR: Relative Risk
SD: Standard Deviation
TCEQ: Texas Commission on Environmental Quality
TDSHS: Texas Department of State Health Services
US: United States
